# Spatiotemporal evolution of online attention to vaccines since 2011: An empirical study in China

**DOI:** 10.3389/fpubh.2022.949482

**Published:** 2022-07-26

**Authors:** Feng Hu, Liping Qiu, Wei Xia, Chi-Fang Liu, Xun Xi, Shuang Zhao, Jiaao Yu, Shaobin Wei, Xiao Hu, Ning Su, Tianyu Hu, Haiyan Zhou, Zhuang Jin

**Affiliations:** ^1^Global Value Chain Research Center, Zhejiang Gongshang University, Hangzhou, China; ^2^Institute of International Business and Economics Innovation and Governance, Shanghai University of International Business and Economics, Shanghai, China; ^3^Department of Business Administration, Cheng Shiu University, Kaohsiung, Taiwan; ^4^School of Management, Shandong Technology and Business University, Yantai, China; ^5^Business School, Hohai University, Nanjing, China; ^6^London College of Communication, University of the Arts London, London, United Kingdom; ^7^Institute of Spatial Planning & Design, Zhejiang University City College, Hangzhou, China; ^8^Cash Crop Workstation, Shangcheng Bureau of Agriculture and Rural Affairs, Shangcheng, China; ^9^School of MBA, Zhejiang Gongshang University, Hangzhou, China; ^10^School of Information Engineering, Zhengzhou University, Zhengzhou, China; ^11^Institute of Artificial Intelligence and Change Management, Shanghai University of International Business and Economics, Shanghai, China; ^12^Baotou Teachers' College, Inner Mongolia University of Science & Technology, Baotou, China

**Keywords:** vaccine, public health, online attention, spatiotemporal characteristics, GeoDetector

## Abstract

Since the outbreak of Coronavirus Disease 2019 (COVID-19), the Chinese government has taken a number of measures to effectively control the pandemic. By the end of 2021, China achieved a full vaccination rate higher than 85%. The Chinese Plan provides an important model for the global fight against COVID-19. Internet search reflects the public's attention toward and potential demand for a particular thing. Research on the spatiotemporal characteristics of online attention to vaccines can determine the spatiotemporal distribution of vaccine demand in China and provides a basis for global public health policy making. This study analyzes the spatiotemporal characteristics of online attention to vaccines and their influencing factors in 31 provinces/municipalities in mainland China with Baidu Index as the data source by using geographic concentration index, coefficient of variation, GeoDetector, and other methods. The following findings are presented. First, online attention to vaccines showed an overall upward trend in China since 2011, especially after 2016. Significant seasonal differences and an unbalanced monthly distribution were observed. Second, there was an obvious geographical imbalance in online attention to vaccines among the provinces/municipalities, generally exhibiting a spatial pattern of “high in the east and low in the west.” Low aggregation and obvious spatial dispersion among the provinces/municipalities were also observed. The geographic distribution of hot and cold spots of online attention to vaccines has clear boundaries. The hot spots are mainly distributed in the central-eastern provinces and the cold spots are in the western provinces. Third, the spatiotemporal differences in online attention to vaccines are the combined result of socioeconomic level, socio-demographic characteristics, and disease control level.

## Introduction

In the long history of human beings, vaccines have been a powerful weapon to fight against infectious diseases. Many infectious diseases have been controlled or eliminated by vaccination. Vaccination is one of the most effective and cost-effective measures to prevent infectious diseases in humans at present. Through widespread vaccination, smallpox was eradicated globally in 1979, as declared by World Health Organization (WHO). The incidences of polio, plague, measles, rabies, pertussis, tetanus, viral hepatitis B, etc. have also been dramatically reduced by vaccination. Since the outbreak of Coronavirus Disease 2019 (COVID-19), the Chinese government has taken a number of measures to effectively control the pandemic (1, 2). By the end of 2021, China achieved a full vaccination rate of higher than 85%. The Chinese Plan provides an important model for the global fight against COVID-19. Dr. Bruce Aylward, team leader of the WHO-China joint mission on COVID-19, stated that China was taking a rigorous and innovative approach to standard public health measures (3). By using a large-scale science-driven flexible mechanism, China has prevented the spread of COVID-19 and changed its course. Dr. Aylward also suggested China was the only country to have the most experience and achievements in the fight against COVID-19, and that other countries should learn from China's rapid response mechanism (4).

With the rapid development of mobile networks, Internet search data are widely used in epidemiological research with the ultimate goal of informing public health and policy (5, 6). Internet search data not only enable Internet users to obtain the required disease prevention information in a short period of time but also assist the government and medical institutions in market forecasting. The general public has formed the habit of searching the Internet for information about the COVID-19 pandemic, prevention methods, drug purchases, and the effect of vaccines (7–9). Accordingly, there has been an increasing public search for and interest in vaccines, from routine ones, such as HPV (10–18), HIV (19–22), and polio (23–28), to those that are promoted during pandemics, such as SARS (29–32), H1N1 (33–40), and COVID-19 (41–52). How to determine public concerns about vaccines is becoming the focus of research in government and medical system decision-making. A literature review revealed that research on online attention to vaccines (OAV) mainly investigated different vaccine types (10–52), correlation with vaccination rates (10, 23), impact on vaccination (44, 53), and vaccination demand prediction and trend analysis (54–57) based on internet search data in terms of different disciplines and perspectives. The concept of the online attention has been widely used in the research of public health events, such as vaccines, but it is mostly based on a single platform and English cultural background.

However, the above studies are mostly based on Google search data, which are primarily relevant to English-speaking countries. Additional research is required to determine whether their conclusions are applicable to other cultures, backgrounds, and search engines. Due to the inaccessibility of Google Search, Baidu is the primary search engine used in China. In 2019, Baidu ranked first in China with a penetration rate of 90.9% (58). Hence, Baidu search data better reflect the public need for vaccines in China than other search engines. Baidu Index shows the changes in the search volume of a particular keyword over time, and directly and objectively reflects the interests and needs of Chinese internet users. Baidu has become one of the most important online platforms for statistical analysis in China (59). As of March 2021, Baidu ranked first in the three major search engine markets (all-platform, PC, and mobile) in China (it had a 70.18% share of the Chinese search engine market in March 2021). Therefore, Baidu Index is reliable. Infodemiology studies based on the Baidu Index platform mainly involve the application in COVID-19 prediction and monitoring (60, 61) but have not investigated OAV. In terms of research content, few studies have analyzed the spatial characteristics of OAV and their causes. Therefore, exploring the spatial characteristics of OAV, the influencing factors, and their effects using Baidu Index data will enable the identification of problems from a new perspective and help to understand and manage public health behaviors in the long term. Internet searches using keywords like vaccines reveal the attention of internet users to vaccines, reflect local awareness and needs for vaccines, and provide a basis for global public health policy making.

Accordingly, this study aims to analyze OAV in 31 provinces/municipalities in China (excluding Hong Kong, Macau, and Taiwan) with Baidu Index as the data source and using measures, such as geographic concentration index and coefficient of variation. Moreover, factors affecting OAV in each province/municipality in 2011, 2017, and 2020 were investigated using indicators, such as GDP per capita and urbanization rate.

## Data and methods

### Data

Just like Google Trends, Baidu Index follows certain rules for calculation and retrieval (62). Data collected from Baidu Index is standardized. It allows researchers to select data from different geographic regions, across gender and other characteristics, during a defined sampling time frame.

To examine the Chinese people's attention to vaccines, data for this study are from Baidu Index (https://index.baidu.com/). Searches using the keyword “vaccine” on https://www.aizhan.com/ and https://www.chinaz.com/ revealed that search terms with the highest search volumes were “vaccine,” “COVID-19 vaccine,” “HPV vaccine,” “9-valent vaccine,” and “vaccination.” At the same time, we took into account that Baidu Index included mobile data in its calculation in 2011, therefore, using these terms as the keywords, the internet search index for the vaccine from January 1, 2011 to December 31, 2021 was calculated to reflect the degree of online attention of interested people/potentially interested people and its changes.

However, keyword data are time sensitive. For example, data on “The COVID-19 vaccine” were not available until February 24, 2020. Therefore, the data sampling period for the empirical part of this study is between February 24, 2020 and December 31, 2021. The same approach was adopted for the data sampling period of the remaining vaccine keywords.

The Baidu Index data revealed a significant trend: Chinese people were more concerned about vaccination status in China. Global vaccination status had no significant impact on searches in China. For example, Baidu Index did not increase significantly on September 21, 2020, when the WHO announced the global COVID-19 vaccine program, COVAX. At that time, vaccination had achieved great success in China, which was significantly different from the situation in other countries.

In terms of research data on influencing factors, GDP size, GDP per capita, urbanization rate, age structure, education, year-end permanent population, sex ratio, infectious disease incidence, and infectious disease mortality were selected as the influencing factors. Data on infectious disease incidence, infectious disease mortality, and sex ratio were from the China Health Statistical Yearbook; data on sex ratio were from 2010, 2016, and 2020 census; and data on the remaining influencing factors were from the China Statistical Yearbook.

### Methods

The spatiotemporal differences in OAV in China since 2011 were assessed using five measures, including coefficient of variation (CV), Herfindahl index (H), primacy index (P), geographic concentration index (G), and seasonal concentration index (S). Influencing factors were analyzed using GeoDetector.

Coefficient of variation (CV) is the ratio of the standard deviation to the mean and shows the degree of variability among the sample measures.


$$\begin{array}{l} CV=\sqrt{\frac{\overset{n}{\underset{i=1}{\sum }}\left(x_{i}-\bar{x}\right)}{n}/\bar{x}} \end{array}$$

where *CV* is the degree of variability in OAV among regions. The larger the *CV*, the more significant the spatial variability in OAV.

Herfindahl index (H) is a comprehensive measure of concentration, reflecting the degree of agglomeration of the regional economic scale. Its value ranges from 0 to 1. The closer it is to 0, the lower the degree of regional economic agglomeration.


$$\begin{array}{l} H=\overset{n}{\underset{i=1}{\sum }}P_{i}^{2} \end{array}$$

where *P*_*i*_ is the ratio of the index of a particular location to the total number. The closer *H* is to 1, the higher the regional agglomeration of OAV.

Primacy index (P) is a relevant measure in economies of scale and agglomeration and is measured by the ratio of the economic scale of the first-ranked region to that of the second-ranked region, reflecting the degree of agglomeration of regional economies.


$$\begin{array}{l} P=P_{1}/P_{2} \end{array}$$

where *P*_1_ and *P*_2_ are the OAV in the regions with the first and second largest scales, respectively. It aims to analyze the concentration of OAV. The larger the *P*, the more concentrated and uneven the OAV is.

The geographic concentration index (G) is a measure of the geographical concentration of economic activity. It reflects the regional concentration or dispersion of OAV. The closer the G is to 100, the more concentrated the OAV is in a particular region; otherwise, the more dispersed it is.


$$\begin{array}{l} G=100\sqrt{\Sigma_{i=1}^{n}{\left(P_{j}/P\;\right)}^{2}} \end{array}$$

where *P*_*j*_ is the OAV in region *j*, and *P* is the total OAV.

Seasonal concentration index (S) reflects the temporal concentration of OAV.


$$\begin{array}{l} S=\sqrt{\overset{12}{\underset{i=1}{\sum }}{\left(x_{i}-8.33\right)}^{2}/12} \end{array}$$

where *x*_*i*_ is the ratio of the monthly to annual total OAV. The larger the *S*, the more uneven the distribution throughout the year, and the greater the temporal variability in OAV, and vice versa.

Global spatial autocorrelation (Moran's I) is used to test the spatial distribution of OAV among provinces/municipalities in China. It is calculated as follows:


$$\begin{array}{l} I=\frac{\overset{n}{\underset{i}{\sum }}\overset{n}{\underset{j\neq i}{\sum }}W_{ij}}{S^{2}\overset{n}{\underset{i}{\sum }}\overset{n}{\underset{j\neq i}{\sum }}W_{ij}} \end{array}$$

where $S^{2}=\frac{1}{n}\overset{n}{\underset{i}{\sum }}{\left(x_{i}-\bar{x}\right)}^{2}$, *x*_*i*_ is the attribute value at *i*, *x* is the arithmetic mean of *x*_*i*_, and *W*_*ij*_ is the spatial weight matrix.

For hot and cold spot analysis (Getis-Ord G_*i*_), according to Getis and Ord (63), the G*i* statistic for each area unit *i* is:


$$\begin{array}{l} G_{i}=\frac{\underset{j}{\sum }w_{ij}}{\underset{j}{\sum }x_{j}} \end{array}$$

where *w*_*ij*_ is the spatial weight value (the spatial weight matrix using the same determination method and specific form as above in Moran's I), and x_*j*_ is the observed value of unit *j*.

GeoDetector is a spatial analysis model used to assess the relationship between a geographical attribute and its explanatory factors (64). It is widely used to investigate the influencing factors of natural and socioeconomic phenomena. GeoDetector requires only a few preconditions and has obvious advantages when dealing with mixed-type data. The factor detector in GeoDetector was used to assess the explanatory power of each influencing factor and its changes in the evolution of OAV. The factor detector is expressed as follows:


$$\begin{array}{l} q=1-\frac{\overset{L}{\underset{h=1}{\sum }}\sigma_{h}^{2}N_{h}}{N\sigma^{2}} \end{array}$$

where *q* is the detection capability of an influencing factor for OAV; h = 1... *L* is the classification of each factor of the variable; *L* is the number of secondary provinces/municipalities; σ^2^ is the variance of OAV in primary provinces/municipalities; $\sigma_{h}^{2}$ is the variance of OAV in secondary provinces/municipalities; *N* is the number of primary provinces/municipalities, and *N*_*h*_ is the number of secondary provinces/municipalities. The value range of *q* is [0, 1]. The larger the *q*, the greater the influence of this factor on OAV in each province/municipality.

## Spatiotemporal evolution of OAV

### Temporal evolution of OAV

**Figure 1:**
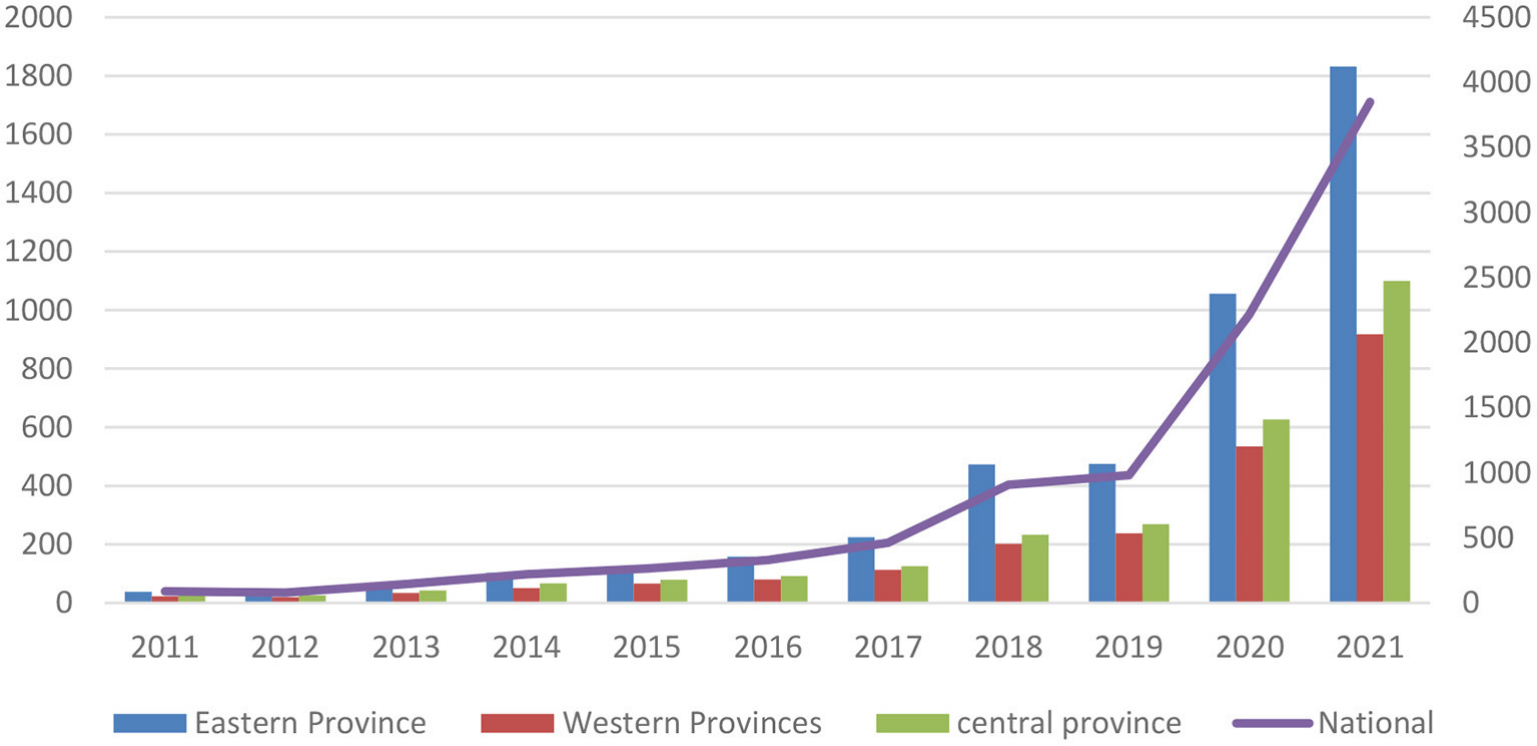
Changes in OAV from 2011 to 2019 (unit: 10,000).

As shown in Figure 1, the total OAV in China increased dramatically from only 890,000 in 2011 to 38,487,600 in 2021, exhibiting a general upward trend. Since 2013, a series of vaccine scandals, such as illegal vaccines in Shandong, and falsification of rabies vaccine production records, had drawn public attention to vaccines. Such attention began to increase significantly in 2016 when the HPV vaccine for cervical cancer prevention was approved in China. There had been a dramatic increase in OAV since 2019 due to the COVID-19 pandemic. Moreover, OAV in the eastern region increased from 379,700 in 2011 to 18,317,000 in 2021, always higher than the national level and those in the central and western regions. OAV in the central region was slightly higher than that in the western region. It generally exhibited a decreasing trend from the eastern to central to western regions.

The ratios of OAV of each month to the monthly average were calculated in Table 1. Except for a few years, the ratios for March, May, July, August, and December were all >1. In particular, the ratio for July was 1.20, and that for August was 1.15. These 5 months can be considered the peak season for vaccine searches. The ratios for January and February were between 0.7 and 0.9, and these 2 months are thus defined as the off-season for vaccine searches. The ratios for April, June, September, October, and November were between 0.9 and 1, and these 5 months are regarded as the shoulder season for vaccine searches. The seasonal concentration index of OAV was >4 from 2011 to 2021, with an average of 5.73, indicating uneven monthly distribution and obvious seasonal differences in OAV in China. The monthly data were aggregated into quarterly data according to the conventional definition of seasons to analyze the quarterly distribution of OAV. In the 2011–2021 period, the indices of OAV in spring, summer, autumn, and winter were 1966.06, 2706.84, 2664.12, and 2339.34, respectively. Summer and autumn are the seasons with higher OAV. Between 2015 and 2019, the average Herfindahl index of national OAV was <0.1, which once again demonstrates the uneven monthly distribution and significant seasonal differences in OAV. In addition, it is worth noting that the month-on-month growth rates in 2020 are larger than those in 2019 due to the COVID-19 pandemic.

**Table 1:** OAV in each month in China between 2011 and 2019 (unit: 10,000).

**Month**	**2011**	**2012**	**2013**	**2014**	**2015**	**2016**	**2017**	**2018**	**2019**	**2020**	**2021**
January	6.1276	4.9510	5.9715	16.4322	20.0434	24.9498	25.8065	44.9147	86.4358	105.1819	288.0900
February	6.5009	7.0802	4.9862	15.5325	16.2985	19.6666	29.1536	35.7974	76.8815	91.2497	192.8527
March	8.9300	8.1371	6.4715	18.4995	22.0646	73.2747	36.4584	56.2070	105.0223	145.2502	360.8367
April	8.4635	7.5255	6.9264	17.2114	21.9430	32.5817	34.1198	51.9747	96.6469	220.1395	364.6814
May	8.7205	7.7090	6.4421	18.1131	23.3494	23.2284	36.5213	82.6084	91.4421	210.4564	463.0280
June	6.9701	6.6224	10.2082	18.4731	22.3731	18.0805	37.1406	68.8988	70.5800	184.2741	429.3844
July	7.1055	6.6405	17.8827	18.9855	23.3171	27.6589	41.0535	195.5302	83.7499	186.6123	396.2210
August	7.5100	6.6981	16.0618	20.8088	25.0268	22.4641	61.3631	83.2805	112.8623	208.6294	361.8486
September	7.2869	6.0434	16.9765	19.7048	23.2770	20.3736	43.4552	72.6461	86.0873	194.4381	242.5200
October	7.5118	6.3923	15.8647	20.7279	23.1627	21.4055	40.2597	73.2803	62.5353	192.8964	245.6573
November	7.4786	6.1858	15.5996	19.9718	22.0601	22.2290	41.8450	81.6458	57.2416	197.4531	272.7819
December	7.0885	5.8339	22.2645	18.0137	24.9450	27.7352	43.5862	79.5985	68.6763	313.1543	274.2530
Seasonal concentration index	4.0275	4.2014	10.4125	3.9040	4.0846	6.3835	4.9364	7.7947	4.8419	6.8597	5.5764
Herfindahl index	0.0843	0.0847	0.1015	0.0839	0.0842	0.1052	0.0872	0.1043	0.0865	0.0904	0.0884

### Spatial differences in OAV

As shown in Table 2, there were obvious geographical imbalances in OAV in China. The highest OAV was measured in Guangdong, Jiangsu, Zhejiang, Beijing, Shandong, and Shanghai in the eastern region, and Henan in the central region. In 2011, the top 10 provinces/municipalities in terms of OAV were Beijing, Shandong, Guangdong, Zhejiang, Henan, Shanghai, Jiangsu, Hebei, Hubei, and Shaanxi, accounting for 47.33% of the national total. In 2016, the top 10 provinces/municipalities were Guangdong, Beijing, Zhejiang, Jiangsu, Shanghai, Sichuan, Shandong, Henan, Hubei, and Fujian, accounting for 53.74% of the national total. In 2021, the top 10 provinces/municipalities were Guangdong, Jiangsu, Shandong, Zhejiang, Beijing, Henan, Sichuan, Hebei, Shanghai, and Anhui, accounting for 54.21% of the national total. The proportion of the top 10 provinces/municipalities in 2021 was greater than those in 2011 and 2016, indicating increasing regional differences in OAV in China.

**Table 2:** OAV in each province/municipality between 2011 and 2021 (unit: 10,000).

**Province/municipality**	**2011**	**2012**	**2013**	**2014**	**2015**	**2016**	**2017**	**2018**	**2019**	**2020**	**2021**	**Total**
Anhui	3.02	2.70	4.56	7.47	8.80	10.14	14.36	29.13	35.28	83.81	143.82	350.59
Beijing	4.77	4.61	8.98	13.24	14.86	20.57	27.47	65.34	57.21	132.97	201.91	561.52
Fujian	3.37	3.10	6.02	9.04	10.32	12.52	16.59	29.78	32.68	71.50	125.18	326.82
Gansu	1.93	1.74	2.43	3.69	4.67	5.33	7.80	13.41	15.94	35.76	64.85	161.74
Guangdong	4.67	4.21	9.91	15.45	19.18	28.58	40.15	85.65	83.41	184.65	347.54	838.68
Guangxi	2.71	2.11	3.99	5.70	7.49	7.99	10.66	19.23	22.97	49.95	95.54	233.82
Guizhou	1.59	1.14	2.38	3.38	5.45	6.43	9.33	15.76	19.17	41.19	74.09	184.15
Hainan	1.11	0.81	1.39	2.26	3.64	4.38	6.54	12.77	13.49	25.91	42.70	117.58
Hebei	3.98	3.62	5.90	8.83	10.32	11.80	15.13	31.64	33.47	91.95	163.17	388.90
Henan	4.26	3.81	6.35	9.90	11.49	13.95	17.51	35.19	42.46	102.14	192.82	449.82
Heilongjiang	2.86	2.52	3.75	5.63	7.20	7.70	10.42	18.20	20.50	52.00	85.27	221.64
Hubei	3.55	3.16	5.93	8.87	10.73	13.20	17.87	32.44	37.00	82.96	132.39	355.22
Hunan	3.37	2.81	5.45	8.68	10.10	11.39	17.11	30.23	33.48	71.00	136.32	336.69
Jilin	2.56	2.33	3.42	4.93	6.41	7.50	10.03	17.56	20.29	45.89	76.71	202.29
Jiangsu	4.04	3.65	7.77	12.14	14.34	18.91	31.01	62.87	65.93	136.45	255.40	625.01
Jiangxi	2.50	2.45	3.84	6.37	7.69	8.61	12.81	22.78	26.76	59.12	102.73	261.52
Liaoning	3.46	2.88	4.93	7.86	8.92	10.20	14.28	26.89	29.01	71.05	121.78	308.89
Inner Mongolia	1.82	1.57	2.64	3.91	5.39	5.94	8.36	14.89	17.60	41.70	71.05	179.10
Ningxia	0.64	0.43	0.75	1.08	1.52	2.12	3.90	6.54	8.16	19.07	31.74	77.85
Qinghai	0.37	0.33	0.57	0.76	0.97	1.70	3.22	6.06	7.63	16.60	28.09	67.93
Shandong	4.70	4.12	7.71	11.54	12.69	16.11	19.82	45.10	49.35	127.13	228.85	539.00
Shanxi	3.15	2.85	4.40	6.84	7.61	8.74	11.01	20.47	24.29	58.76	107.86	262.21
Shaanxi	3.55	3.15	5.24	7.91	9.15	10.76	14.22	25.49	31.53	66.95	121.42	304.69
Shanghai	4.13	3.75	7.37	11.05	13.11	17.73	26.88	55.39	48.93	103.70	160.37	461.51
Sichuan	3.21	3.09	6.12	9.05	11.45	16.63	20.85	40.42	45.51	105.97	163.86	434.55
Tianjin	2.72	2.50	4.41	6.73	7.66	8.38	10.77	20.89	22.61	49.68	77.88	218.54
Tibet	0.20	0.15	0.19	0.28	0.34	0.64	1.62	3.63	4.82	11.76	19.47	44.35
Xinjiang	1.88	1.39	2.03	3.24	4.32	5.22	6.94	11.86	14.19	37.71	55.97	148.32
Yunnan	2.20	2.03	3.52	5.65	6.91	7.72	11.33	18.77	21.44	49.97	95.79	230.68
Zhejiang	4.47	4.25	8.41	12.62	14.28	19.02	30.01	63.53	67.41	132.16	228.71	595.63
Chongqing	2.21	2.06	3.84	6.15	8.13	9.89	14.55	25.52	28.39	58.28	95.51	259.66

To describe the regional differences in OAV among the 31 provinces/municipalities in mainland China, a comprehensive analysis was conducted using the coefficient of variation, Herfindahl index, geographic concentration index, and primacy index (Table 3).

**Table 3:** Differences in OAV among provinces/municipalities and among the three regions in China from 2011 to 2021.

**Year**	**Differences among**				**Differences among**			
	**provinces/municipalities**				**three regions**			
	**CV**	**P**	**G**	**H**	**CV**	**P**	**G**	**H**
2011	0.4339	1.0156	19.5774	0.0383	0.2165	1.3221	59.0732	0.3490
2012	0.4618	1.0829	19.7854	0.0391	0.2399	1.3576	59.3726	0.3525
2013	0.5283	1.1037	20.3115	0.0413	0.3010	1.5916	60.2934	0.3635
2014	0.5268	1.1666	20.2974	0.0412	0.2973	1.5464	60.2321	0.3628
2015	0.4935	1.2909	20.0271	0.0401	0.2632	1.5248	59.7016	0.3564
2016	0.5688	1.3896	20.6636	0.0427	0.3120	1.7281	60.4794	0.3658
2017	0.5790	1.2948	20.7532	0.0431	0.3236	1.7891	60.6819	0.3682
2018	0.6585	1.3108	21.5041	0.0462	0.4008	2.0309	62.1995	0.3869
2019	0.5803	1.2373	20.7651	0.0431	0.3215	1.7634	60.6450	0.3678
2020	0.5615	1.3533	20.5985	0.0424	0.3073	1.6851	60.3994	0.3648
2021	0.5836	1.3608	20.7952	0.0432	0.3080	1.6656	60.4112	0.3650

Large spatial differences with unstable fluctuations were found in OAV in the 2011–2021 period. The coefficient of variation in the 10-year period was approximately 0.54 on average and increased ear by year. The Herfindahl index was close to 0 and showed small fluctuations, indicating low agglomeration and obvious spatial dispersion of OAV among the various provinces/municipalities. The primacy index remained <2 and fluctuated slowly, and the geographic concentration index was also small, indicating moderate agglomeration and normal spatial structure of OAV among the various provinces/municipalities.

Moreover, there were spatial differences in OAV among the eastern, central, and western regions in the 2011–2021 period. The coefficient of variation maintained a fluctuating growth year by year, with the lowest degree of difference among the three regions in 2011. The Herfindahl index was approximately 0.36 and generally increased year by year, indicating a spatial concentration trend of OAV. The primacy index remained <2 and increased year by year, indicating that there was an increasing difference in OAV between the first and second-ranked regions. The average geographical concentration index was 60.37, and the gap was widening. It suggests that OAV was increasingly concentrated in a particular region from 2011 to 2021.

Furthermore, Table 4 was prepared to measure OAV within the eastern, central, and western regions in mainland China. The comparison revealed obvious spatial differences in OAV within eastern, central, and western China in the 2011–2021 period. The coefficient of variation generally showed a decreasing trend from the western to eastern to central regions. It indicates that the spatial difference in OAV was relatively large in the western region and relatively small in the central region and that internet users in all provinces/municipalities in the eastern region generally paid close attention to vaccines. From 2011 to 2021, the Herfindahl index in all three regions was >0.1 and fluctuated only slightly. It suggests that OAV was dispersed within each region, without excessive aggregation in one or a few provinces/municipalities. Geographic concentration indices in the three regions fluctuated slightly between 32 and 34 in the 2011–2021 period, indicating a low agglomeration and spatial dispersion of OAV in each region. In the 10-year period, the primacy indices of OAV in all three regions were >1 and remained stable. It suggests that there were small differences between the first and second-ranked provinces/municipalities, indicating a low agglomeration and balanced spatial structure of OAV within each region.

**Table 4:** Intra-regional differences in OAV from 2011 to 2021.

**Year**	**CV**			***P***			**G**			**H**		
	**East**	**West**	**Central**	**East**	**West**	**Central**	**East**	**West**	**Central**	**East**	**West**	**Central**
2011	0.2852	0.5420	0.1617	1.0156	1.2861	1.1658	32.8838	32.8356	33.7662	0.1081	0.1078	0.1140
2012	0.3053	0.5893	0.1479	1.0829	1.2991	1.1928	33.0641	33.5074	33.6957	0.1093	0.1123	0.1135
2013	0.3472	0.6249	0.2016	1.1037	1.3924	1.2425	33.4742	34.0406	34.0036	0.1121	0.1159	0.1156
2014	0.3465	0.6264	0.2061	1.1666	1.3264	1.2461	33.4675	34.0637	34.0339	0.1120	0.1160	0.1158
2015	0.3390	0.5927	0.1844	1.2909	1.3057	1.2296	33.3900	33.5574	33.8956	0.1115	0.1126	0.1149
2016	0.4113	0.6327	0.2144	1.3896	1.5891	1.3278	34.1930	34.1609	34.0906	0.1169	0.1167	0.1162
2017	0.4396	0.5535	0.2086	1.2948	1.3880	1.2183	34.5441	32.9947	34.0511	0.1193	0.1089	0.1159
2018	0.4639	0.5837	0.2330	1.3108	1.5838	1.4256	34.8601	33.4260	34.2265	0.1215	0.1117	0.1171
2019	0.4379	0.5547	0.2425	1.2373	1.5686	1.3867	34.5221	33.0119	34.2994	0.1192	0.1090	0.1176
2020	0.4229	0.5484	0.2414	1.3533	1.5489	1.2644	34.3347	32.9234	34.2907	0.1179	0.1084	0.1176
2021	0.4628	0.5196	0.2697	1.3608	1.3495	1.3407	34.8446	32.5325	34.5246	0.1214	0.1058	0.1192

Global Moran's I was used to preliminarily examine the spatial agglomeration characteristics of OAV, and very significant results (Moran's I = 0.119711, Z-score = 2.858161, *p* = 0.004261) were obtained, indicating a strong spatial autocorrelation for OAV. It fully demonstrates the spatial characteristics of high and low-value agglomeration of OAV in China.

**Figure 2:**
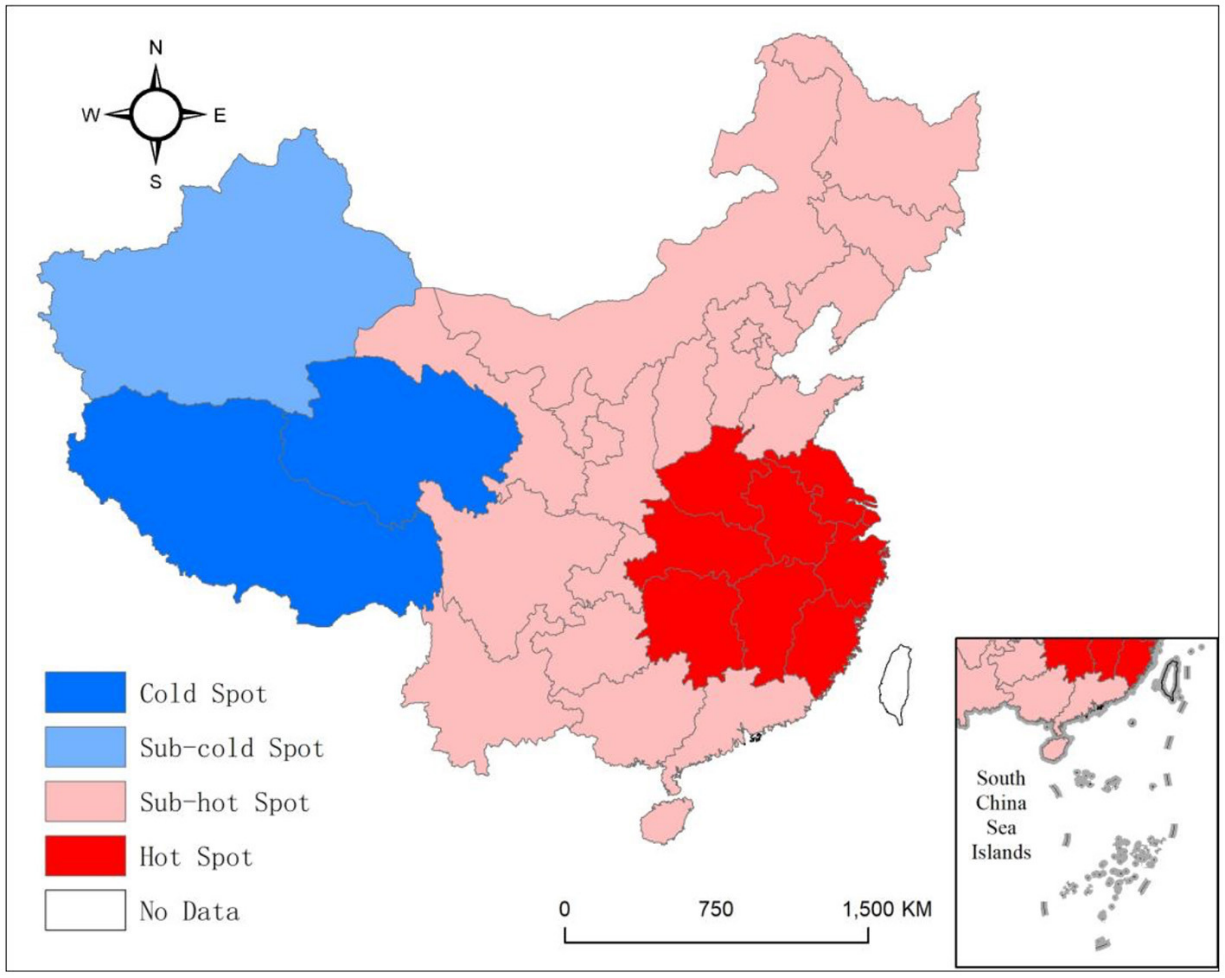
Distribution of hot and cold spots of OAV in China.

The distribution of hot and cold spots of OAV in China was determined by using the *G*_*i*_ statistic to detect local hot spots. The *G*_*i*_ statistic was divided into four levels from high to low by Jenks's natural breaks (Figure 2). The results show that hot and cold spots are distributed in east and west China, respectively, and have clear boundaries. The hotspots are mainly distributed in the central-eastern coastal provinces, of which the Yangtze River Delta provinces are the core hot spot area. The remaining central-eastern region is the sub-hot spot area. The average OAV was 3,060,700 in this region. The eastern coastal region is developed in the traditional sense, where China's three major urban agglomerations are located. On the one hand, this region has a large number of permanent residents and a dense population, and on the other hand, it has a high level of education, thus resulting in high demand and awareness of vaccines. The cold spots are distributed in the western region, including Xinjiang, Tibet, and Qinghai. The average OAV was 868,700. Specifically, Tibet and Qinghai are the core cold spot area, and Xinjiang is the sub-cold spot area. The western region, especially the northwest region, is relatively insensitive to vaccines due to its deep inland location and low population density. On the whole, the spatial distribution of OAV can be preliminarily attributed to the socioeconomic conditions and population size based on the differences in the distribution of hot and cold spots between east and west China. In other words, socioeconomic conditions and population size promote OAV to a certain extent.

## Influencing factors of OAV

### Selection of influencing factors of OAV

Considering comprehensiveness and data availability, and due to the fact that the influencing factor indicators in 2021 have not yet been published, provincial/municipal OAV in 2011, 2017, and 2020 was used as the explained variable. In particular, 2017 was also selected to detect the changes in influencing factors after the approval of the HPV vaccine for cervical cancer prevention. The correlation coefficients between OAV and its influencing factors were calculated using 13 explanatory variables, such as GDP, per capita GDP, urbanization rate, and infectious disease incidence. Before employing the GeoDetector, the independent variables were stratified by natural breaks using ArcGIS. Each influencing factor was divided into five classes. Consistent classification criteria were applied at different stages. Finally, the GeoDetector was used to measure the influence of each influencing factor on OAV. The results are shown in Table 5.

**Table 5:** Correlation coefficient between OAV and its influencing factors.

**Influencing factor**			**Correlation**			**Driving effect**
			**2011**	**2017**	**2020**	
Socioeconomic level	GDP size		0.8391***	0.7590***	0.8753***	↑
	GDP per capita		0.3738	0.5431**	0.4199	↑
	Urbanization rate		0.3809	0.3661	0.2572	↓
Socio-demographic characteristics	Age structure	0–14 years	0.3524	0.3980	0.4323	↑
		15–64 years	0.6188***	0.5181**	0.6427***	↑
		65 years or older	0.6285***	0.4874**	0.5876***	↓
	Education	Junior college or above	0.7786***	0.4819**	0.6409***	↑
		High school	0.3658	0.2601	0.4093	↑
		Junior high school	0.3134	0.2799	0.4206	↑
	Year-end population		0.6085***	0.5242**	0.6404***	↑
	Sex ratio		0.1258	0.0733	0.0826	↓
Level of disease control	Infectious disease incidence		0.9007***	0.8634***	0.9087***	↑
	Infectious disease mortality		0.0671	0.0456	0.0694	↑

^**^, ^***^Significance at the 5, and 1% levels, respectively.

↑The upward arrow represents the correlation between vaccine network attention and a certain influencing factor, which is numerically larger in 2020 than in 2011 or 2017.

↓The downward arrow represents the correlation between vaccine network attention and a certain influencing factor, which is numerically smaller in 2020 than in 2011 or 2017.

### Result analysis of influencing factors of OAV

The GeoDetector results show that only 6–7 of the 13 factors have an influence on the spatial distribution of OAV, indicating that the distribution of OAV is affected by various factors, such as the economic strength and level of disease control of the provinces/municipalities. The explanatory power of each influencing factor is between 40 and 90%. They can be divided into primary and secondary influencing factors according to the explanatory power. The primary influencing factors include infectious disease incidence and GDP size. Secondary influencing factors include the number of people aged 15–64, the number of people aged 65 and above, the number of people in junior college or higher education, and the year-end population.

#### Level of disease control

The incidence of infectious diseases has the most significant influence on the spatial distribution of OAV. q values were at least as high as 0.86, indicating that the incidence of infectious diseases has a great influence on OAV in each province/municipality. Infectious diseases pose a great threat to public health. Vaccination is the most cost-effective and effective public health intervention for the prevention and control of infectious diseases. For families, it is also an effective means to reduce the incidence of diseases and consequent medical expenses. The incidence of infectious diseases (1/100,000) in China decreased from 2139.69 in 1955 to 190.36 in 2020 as a result of vaccination. The mortality (1/100,000) also decreased from 18.43 in 1955 to 1.87 in 2020. The influence of infectious disease incidence on OAV in 2017 (0.8634) decreased compared with 2011 but increased again in 2020, which might be due to the outbreak of COVID-19.

#### Socioeconomic level

The influence of GDP size on OAV is self-evident. The greater the GDP size, the more developed the urban infrastructure and services, and the higher the level of informatization. The regression results revealed that except for 2017, the *q-*values were all >0.8. The more developed a region is, the better the public understanding of vaccines, and the better the health services and facilities, leading to higher public attention to vaccines. The influence of GDP size on OAV increased significantly in 2020 as compared to 2011 to 2017, which may be related to the fact that most COVID-19 cases occurred in relatively developed regions. GDP per capita reflects the local per capita income level to a certain extent. This influencing factor was only significant in regression analysis for 2017, which may be related to the approval of the HPV vaccine for cervical cancer prevention. The total HPV vaccination costs ranged from more than one thousand yuan to even tens of thousands of yuan which included extra costs incurred due to its scarcity. This is a high cost and thus people with higher incomes are more likely to consider vaccination. In recent years, many provinces/municipalities have provided free vaccination for females of the appropriate age, thus making this influencing factor insignificant. The influence of the urbanization rate was not significant in regression analysis, and the *q*-value decreased year by year. This result is closely related to the improvement of rural infrastructure and the popularization of the Internet in the process of rural revitalization in China in recent years. According to the *48th Statistical Report on the Development of Internet in China*, as of June 2021, the Internet penetration rate in China's urban and rural areas was 78.3 and 59.2%, respectively, and the gap in Internet penetration between urban and rural areas is narrowing year by year.

#### Socio-demographic characteristics

In terms of age structure, the influence of children aged 0–14 was not significant. This may be related to China's childhood immunization program, which implements free and mandatory vaccination for children at this age stage. The influences on people aged 15–64 and those aged 65 and above were not significant. One possible reason is that these groups already have some understanding of vaccines. Another possible reason is that according to the *48th Statistical Report on the Development of Internet in China*, more than 80% of Chinese internet users are over the age of 20. Hence, these groups also constitute the main body of internet users. A significant influence was observed for people with the junior college or higher education, indicating that OAV in each province/municipality in China was indeed affected by local education levels. The regression results from GeoDetector for the year-end population were also significant, indicating that the population size of provinces/municipalities also influenced OAV and that this influence increased year by year.

## Conclusions and implications

### Conclusions

This study investigated the characteristics and spatial evolution of OAV in 31 provinces/municipalities in China since 2011 by spatial data analysis and analyzed the influencing factors by GeoDetector. The following conclusions are drawn:

First, the overall OAV in China showed a rising trend from 2011 to 2021, especially after 2016 due to the HPV vaccines for cervical cancer prevention and the COVID-19 vaccines. The peak season for OAV is May, December, March, August, and July. The monthly distribution of OAV in China was uneven, with obvious seasonal differences.

Second, in the 2011–2021 period, there were significant differences in OAV among provinces/municipalities in China, exhibiting a spatial pattern of “high in the east and low in the west.” The highest OAV was measured in Guangdong, Jiangsu, Zhejiang, Beijing, Shandong, and Shanghai in the eastern region, and Henan in the central region. There were large spatial differences with unstable fluctuations, low agglomeration, and obvious dispersion of OAV among provinces/municipalities in the 2011–2021 period. There was an increasing spatial concentration trend of OAV among the eastern, central, and western regions, and an increasing difference between the first and second-ranked regions in the 2011–2021 period.

Third, there were differences in OAV within the eastern, central, and western regions. From the perspective of inter-regional differences, the coefficient of variation generally showed a decreasing trend from the western to eastern to central regions. The Herfindahl index in all three regions was >0.1 and fluctuated only slightly, suggesting that there was no excessive aggregation of OAV in one or a few provinces/municipalities. The primacy indices of OAV in all three regions were >1 and remained stable. The geographic concentration index fluctuated between 32 and 34. It suggests that there were small differences between the first and second-ranked provinces/municipalities, indicating a dispersion, low agglomeration, and balanced spatial structure of OAV within each region.

Fourth, OAV in China showed the spatial characteristics of high and low-value agglomeration. The hotspots are mainly distributed in the central-eastern coastal provinces, of which the Yangtze River Delta provinces are the core hot spot area. The remaining central-eastern region is the sub-hot spot area. The cold spots are distributed in the western region. The spatial distribution of OAV can be preliminarily attributed to the socioeconomic conditions and population size. In other words, socioeconomic conditions and population size promote OAV to a certain extent.

Fifth and lastly, the GeoDetector analysis shows that socioeconomic level, socio-demographic characteristics, and disease control level are the main factors influencing the spatiotemporal differences in OAV. Specifically, the primary influencing factors include infectious disease incidence and GDP size. Secondary influencing factors include the number of people aged 15–64, the number of people aged 65 and above, the number of people in junior college or higher education, and the year-end population.

### Implications and suggestions

The results of this study provide some implications for policy-makers.

First, vaccine awareness is generally low in remote areas, such as western China. Local governments need to improve the public's knowledge of infectious diseases and the benefits of vaccination to increase the vaccination rate in appropriate age groups. In particular, given that the COVID-19 pandemic has not ended, much still remains to be done to promote COVID-19 vaccination. Although remote areas are sparsely populated, there is still a great risk of COVID-19 exposure from other areas. Vaccination is the most effective measure to control the COVID-19 pandemic at present. There is an urgent need to raise public awareness on vaccination for the prevention of COVID-19.

Second, some vaccines, such as HPV vaccines, may have high costs. Wherever possible, local governments should try their best to offer convenient medical insurance at a low price, and reduce the cost of vaccination by measures such as medical insurance funds or partner assistance to reduce the incidence of infectious diseases.

Third and last, there are differences between young and middle-aged people and the elderly in the ways and convenience of receiving information. Hence, different measures should be implemented for different age groups for publicity of vaccines and infectious diseases. For example, offline publicity can be given to the elderly through senior activity centers.

Despite its contribution, this study has some limitations. On the one hand, the Baidu engine is not the only search tool used by Chinese internet users. In recent years, with the rise of large online platforms, such as 360 and Sogou, other search engines have also been used. Therefore, the data used for this study are not comprehensive enough. On the other hand, the influencing factors included in this study are still limited. There may be other influencing factors that need to be addressed. Further research is also required to better understand the mechanism of the influencing factors.

## Data availability statement

The original contributions presented in the study are included in the article/supplementary material, further inquiries can be directed to the corresponding author/s.

## Author contributions

All authors undertook research, writing, and review tasks throughout this study. All authors have read and agreed to the published version of the manuscript.

## Funding

This work was supported by the Major Program of the National Social Science Foundation of China [grant number 20&ZD124], the National Natural Science Foundation of China [grant numbers 72103129, 72173014, 71973129, 72072162, and 71773115], the National Social Science Foundation of China [grant number 21CJY024], the Philosophy and Social Science Program of Zhejiang [grant number 22NDQN290YB], and the Humanity and Social Science Foundation of Ministry of Education of China [grant numbers 21YJA790043, 21YJA630037, 19YJA790107, 18YJA790088, and 21YJCZH213].

## Conflict of interest

The authors declare that the research was conducted in the absence of any commercial or financial relationships that could be construed as a potential conflict of interest.

## Publisher's note

All claims expressed in this article are solely those of the authors and do not necessarily represent those of their affiliated organizations, or those of the publisher, the editors and the reviewers. Any product that may be evaluated in this article, or claim that may be made by its manufacturer, is not guaranteed or endorsed by the publisher.

## References

[1] Hu F Qiu L Xi X Zhou H Hu T Su N. Has COVID-19 Changed China's digital trade?-implications for health economics. Front Publ Health. (2022) 10:831549. 10.3389/fpubh.2022.831549 35309208 PMC8924300

[2] Hu F Shi X Wang H Nan N Wang K Wei S. Is health contagious?-Based on empirical evidence from China family panel studies' data. Front Publ Health. (2021) 9:691746. 10.3389/fpubh.2021.691746 34277551 PMC8283520

[3] Cohen KK. China's aggressive measures have slowed the coronavirus. They may not work in other countries. Science. (2020) 367:727. 10.1126/science.abb542632054740

[4] Aylward B Liang W. Report of the WHO-China joint mission on coronavirus disease 2019 (COVID-19). Geneva: WHO, (2020).

[5] Eysenbach I. Tracking flu-related searches on the web for syndromic surveillance. In: AMIA Annu Symp Proc. (2006) p. 244–8. PMC1839505 17238340

[6] Infodemiology EG. infoveillance: framework for an emerging set of public health informatics methods to analyze search, communication and publication behavior on the Internet. J Med Internet Res. (2009) 11:e11. 10.2196/jmir.1157 19329408 PMC2762766

[7] Xi X Wei S Lin K-L Zhou H Wang K Zhou H. Digital technology, knowledge level, and food safety governance: implications for national healthcare system. Front Publ Health. (2021) 9:753950. 10.3389/fpubh.2021.753950 34900901 PMC8655841

[8] Hu F Xi X Zhang Y. Influencing mechanism of reverse knowledge spillover on investment enterprises' technological progress: an empirical examination of Chinese firms. Technol Forecast Soc Change. (2021) 169:120797. 10.1016/j.techfore.2021.120797

[9] Hu F Qiu L Zhou H. Medical device product innovation choices in Asia: an empirical analysis based on product space. Front Publ Health. (2022) 10:871575. 10.3389/fpubh.2022.871575 35493362 PMC9043244

[10] Kalichman SC and Kegler C. Vaccine-related internet search activity predicts H1N1 and HPV vaccine coverage: implications for vaccine acceptance. J Health Commun. (2015) 20:259–65. 10.1080/10810730.2013.852274 25222149

[11] Simonart T Hoai X-LL De Maertelaer V. Impact of human papillomavirus vaccine in reducing genital warts: a Google trends analysis. J Am Acad Dermatol. (2022) 86:956–8. 10.1016/j.jaad.2021.03.091 33812953

[12] Gollust SE LoRusso SM Nagler RH Fowler EF. Understanding the role of the news media in HPV vaccine uptake in the United States: synthesis and commentary. Hum Vaccines Immunother. (2016) 12:1430–4. 10.1080/21645515.2015.1109169 26554612 PMC4964732

[13] Fu LY Zook K Spoehr-Labutta Z Hu P Joseph JG. Search engine ranking, quality, and content of web pages that are critical versus noncritical of human papillomavirus vaccine. J Adolesc Health. (2016) 58:33–9. 10.1016/j.jadohealth.2015.09.016 26559742 PMC4695228

[14] McRee A-L Reiter PL Brewer NT. Parents' Internet use for information about HPV vaccine. Vaccine. (2012) 30:3757–62. 10.1016/j.vaccine.2011.11.113 22172505 PMC3346865

[15] Nan X Madden K. HPV vaccine information in the blogosphere: how positive and negative blogs influence vaccine-related risk perceptions, attitudes, and behavioral intentions. Health Commun. (2012) 27:829–36. 10.1080/10410236.2012.661348 22452582

[16] Unger Z Maitra A Kohn J Devaskar S Stern L Patel A. Knowledge of HPV and HPV vaccine among women ages 19 to 26. Women's Health Issues. (2015) 25:458–62. 10.1016/j.whi.2015.06.003 26212317

[17] Bynum SA Malo TL Lee J-H Guiliano AR and Vadaparampil ST. HPV vaccine information-seeking behaviors among US physicians: government, media, or colleagues?. Vaccine. (2011) 29:5090–3. 10.1016/j.vaccine.2011.04.134 21619906 PMC3138888

[18] Dib F Mayaud P Longfier L Chauvin P Launay O. Effect of Internet use for searching information on vaccination on the uptake of human papillomavirus vaccine in France: a path-analysis approach. Prevent Med. (2021) 149:106615. 10.1016/j.ypmed.2021.106615 33989671

[19] Mavragani A Ochoa G. Forecasting AIDS prevalence in the United States using online search traffic data. J Big Data. (2018) 5:1–21. 10.1186/s40537-018-0126-7

[20] Ling R Lee J. Disease monitoring and health campaign evaluation using Google search activities for HIV and AIDS, stroke, colorectal cancer, and marijuana use in Canada: a retrospective observational study. JMIR Publ Health Surveill. (2016) 2:e6504. 10.2196/publichealth.6504 27733330 PMC5081479

[21] Jena AB Karaca-Mandic P Weaver L Seabury SA. Predicting new diagnoses of HIV infection using internet search engine data. Clin Infect Dis. (2013) 56:1352–3. 10.1093/cid/cit022 23334812

[22] Mahroum N Bragazzi NL Brigo F Waknin R Sharif K Mahagna H. Capturing public interest toward new tools for controlling human immunodeficiency virus (HIV) infection exploiting data from Google Trends. Health Inform J. (2019) 25:1383–97. 10.1177/1460458218766573 29638172

[23] Layug EJV Espiritu AI Calotes-Castillo LV Jamora RDG. The association of online search interest with polio cases and vaccine coverage: an infodemiological and ecological study. Eur J Pediatr. (2021) 180:2435–41. 10.1007/s00431-021-04049-4 33772622

[24] Orr D Baram-Tsabari A Landsman K. Social media as a platform for health-related public debates and discussions: the Polio vaccine on Facebook. Israel J Health Policy Res. (2016) 5:1–11. 10.1186/s13584-016-0093-4 27843544 PMC5103590

[25] Tur-Sinai A Gur-Arie R Davidovitch N Kopel E Glazer Y Anis E. Socioeconomic status, health inequalities, and solidarity trends in a mass vaccination campaign. Eur J Publ Health. (2019) 29(Suppl. 4):ckz185.404. 10.1093/eurpub/ckz185.404 PMC6628472 31307532

[26] Sagy I Novack V Gdalevich M Greenberg D. Mass media effect on vaccines uptake during silent polio outbreak. Vaccine. (2018) 36:1556–60. 10.1016/j.vaccine.2018.02.035 29439866

[27] Orr D and Baram-Tsabari A. Science and politics in the polio vaccination debate on Facebook: a mixed-methods approach to public engagement in a science-based dialogue. J Microbiol Biol Educ. (2018) 19:19.1.80. 10.1128/jmbe.v19i1.1500 29904532 PMC5969418

[28] Elkin LE Pullon SR and Stubbe MH. 'Should I vaccinate my child?'Comparing the displayed stances of vaccine information retrieved from Google, Facebook and YouTube. Vaccine. (2020) 38:2771–8. 10.1016/j.vaccine.2020.02.041 32107061

[29] Montalti M Rallo F Guaraldi F Bartoli L Po G Stillo M. Would parents get their children vaccinated against SARS-CoV-2? Rate and predictors of vaccine hesitancy according to a survey over 5000 families from Bologna, Italy. Vaccines. (2021) 9:366. 10.3390/vaccines9040366 33920109 PMC8069076

[30] Mayo-Yáñez M González-Torres L Calvo-Henríquez C Chiesa-Estomba C. Estudio de la búsqueda de información sobre la pandemia SARS-CoV-2 en Galicia. Galicia Clin. (2021) 82:13–6. 10.22546/60/2305

[31] Henry BM de Oliveira MHS de Oliveira TB Notarte KI Lippi G. Symptomatology associated with the diffusion of the SARS-CoV-2 Lambda variant in Peru: an infodemiologic analysis. medRxiv Preprint: 21262245. (2021). 10.1101/2021.08.24.21262245

[32] Santangelo OE Provenzano S Gianfredi V. Infodemiology of flu: Google trends-based analysis of Italians' digital behavior and a focus on SARS-CoV-2, Italy. J Prev Med Hyg. (2021) 62:E586–91. 10.15167/2421-4248/jpmh2021.62.3.1704 34909483 PMC8639123

[33] Malik MT Gumel A Thompson LH Strome T Mahmud SM. “Google flu trends” and emergency department triage data predicted the 2009 pandemic H1N1 waves in Manitoba. Can J Publ Health. (2011) 102:294–7. 10.1007/BF03404053 21913587 PMC6974178

[34] Li S Zhou X. Research of the correlation between the H1N1 morbidity data and Google Trends in Egypt. arXiv preprint arXiv:151105300 (2015). 10.48550/arXiv.1511.05300

[35] Cervellin G Comelli I Lippi G. Is Google Trends a reliable tool for digital epidemiology? Insights from different clinical settings. J Epidemiol Glob Health. (2017) 7:185–9. 10.1016/j.jegh.2017.06.001 28756828 PMC7320449

[36] Hill S Mao J Ungar L Hennessy S Leonard CE Holmes J. Natural supplements for H1N1 influenza: retrospective observational infodemiology study of information and search activity on the Internet. J Med Intern Res. (2011) 13:e1722. 10.2196/jmir.1722 21558062 PMC3221378

[37] Zhou X Li Q Zhao H Li S Yu L Tang F. Assessing Google correlate queries for influenza H1N1 surveillance in Asian developing countries. arXiv preprint arXiv:151203132. (2015). 10.48550/arXiv.1512.03132

[38] Chanel O Luchini S Massoni S Vergnaud J-C. Impact of information on intentions to vaccinate in a potential epidemic: swine-origin Influenza A (H1N1). Soc Sci Med. (2011) 72:142–8. 10.1016/j.socscimed.2010.11.018 21163566

[39] Nawa N Kogaki S Takahashi K Ishida H Baden H Katsuragi S. Analysis of public concerns about influenza vaccinations by mining a massive online question dataset in Japan. Vaccine. (2016) 34:3207–13. 10.1016/j.vaccine.2016.01.008 26776467

[40] Cook S Conrad C Fowlkes AL Mohebbi MH. Assessing Google flu trends performance in the United States during the 2009 influenza virus A (H1N1) pandemic. PLoS ONE. (2011) 6:e23610. 10.1371/journal.pone.0023610 21886802 PMC3158788

[41] Pullan S Dey M. Vaccine hesitancy and anti-vaccination in the time of COVID-19: a Google Trends analysis. Vaccine. (2021) 39:1877–81. 10.1016/j.vaccine.2021.03.019 33715904 PMC7936546

[42] Maugeri A Barchitta M Agodi A. Using Google trends to predict Covid-19 vaccinations and monitor search behaviours about vaccines: a retrospective analysis of Italian data. Vaccines. (2022) 10:119. 10.3390/vaccines10010119 35062780 PMC8778420

[43] Khakimova A Abdollahi L Zolotarev O Rahim F. Global interest in vaccines during the COVID-19 pandemic: evidence from Google Trends. Vaccine. (2022) 10:100152. 10.1016/j.jvacx.2022.100152 35291263 PMC8915451

[44] Diaz P Reddy P Ramasahayam R Kuchakulla M Ramasamy R. COVID-19 vaccine hesitancy linked to increased internet search queries for side effects on fertility potential in the initial rollout phase following Emergency Use Authorization. Andrologia. (2021) 53:e14156. 10.1111/and.14156 34181273 PMC8420403

[45] Sycinska-Dziarnowska M Paradowska-Stankiewicz I Wozniak K. The global interest in vaccines and its prediction and perspectives in the era of COVID-19. Real-time surveillance using Google Trends. Int J Environ Res Publ Health. (2021) 18:7841. 10.3390/ijerph18157841 34360134 PMC8345601

[46] Griffith J Marani H Monkman H. COVID-19 vaccine hesitancy in Canada: content analysis of tweets using the theoretical domains framework. J Med Intern Res. (2021) 23:e26874. 10.2196/26874 33769946 PMC8045776

[47] Islam MS Kamal A-HM Kabir A Southern DL Khan SH Hasan SM. COVID-19 vaccine rumors and conspiracy theories: the need for cognitive inoculation against misinformation to improve vaccine adherence. PLoS ONE. (2021) 16:e0251605. 10.1371/journal.pone.0251605 33979412 PMC8115834

[48] An L Russell DM Mihalcea R Bacon E Huffman S Resnicow K. Online search behavior related to COVID-19 vaccines: infodemiology study. JMIR Infodemiol. (2021) 1:e32127. 10.2196/32127 34841200 PMC8601025

[49] Awijen H Zaied YB Nguyen DK. Covid-19 vaccination, fear and anxiety: evidence from Google search trends. Soc Sci Med. (2022) 297:114820. 10.1016/j.socscimed.2022.114820 35183946 PMC8847077

[50] Badell-Grau RA Cuff JP Kelly BP Waller-Evans H Lloyd-Evans E. Investigating the prevalence of reactive online searching in the COVID-19 pandemic: infoveillance study. J Med Intern Res. (2020) 22:e19791. 10.2196/19791 32915763 PMC7595752

[51] Du H Yang J King RB Yang L Chi P. COVID-19 increases online searches for emotional and health-related terms. Appl Psychol. (2020) 12:1039–3. 10.1111/aphw.12237 33052612 PMC7675240

[52] Pascual-Ferrá P Alperstein N Barnett DJ. A multi-platform approach to monitoring negative dominance for COVID-19 vaccine-related information online. Disast Med Publ Health Preparedn. (2021) 1–9. 10.1017/dmp.2021.136 PMC8209443 33938423

[53] Rovetta A. Google Trends as a predictive tool for COVID-19 vaccinations in Italy: retrospective infodemiological analysis. JMIRx Med. (2022) 3:e35356. 10.2196/35356 35481982 PMC9031689

[54] Milinovich GJ Avril SM Clements AC Brownstein JS Tong S Hu W. Using internet search queries for infectious disease surveillance: screening diseases for suitability. BMC Infect Dis. (2014) 14:690. 10.1186/s12879-014-0690-1 25551277 PMC4300155

[55] Shah MP Lopman BA Tate JE Harris J Esparza-Aguilar M Sanchez-Uribe E. Use of internet search data to monitor rotavirus vaccine impact in the United States, United Kingdom, and Mexico. J Pediatr Infect Dis Soc. (2018) 7:56–63. 10.1093/jpids/pix004 28369477 PMC5608630

[56] Rampally V Mondal H Mondal S. Global search trends on common vaccine-related information in English on the Internet. J Fam Med Primary Care. (2020) 9:698. 10.4103/jfmpc.jfmpc_1001_19 32318405 PMC7113945

[57] Index B,. (2020). Available online at: https://index.baidu.com/v2/index.html#/ (accessed March 28, 2020).

[58] Puri N Coomes EA Haghbayan H Gunaratne K. Social media and vaccine hesitancy: new updates for the era of COVID-19 and globalized infectious diseases. Hum Vaccines Immunother. (2020) 16:2586–93. 10.1080/21645515.2020.1780846 32693678 PMC7733887

[59] Mondal S Mondal H Samantaray R. Information-seeking behavior on Coronavirus Disease-19 Vaccine on the internet: a global and Indian search trend analysis. J Sci Soc. (2021) 48:93. 10.4103/jss.jss_18_21

[60] Fang J Zhang X Tong Y Xia Y Liu H Wu K. Baidu index and COVID-19 epidemic forecast: evidence from China. Front Publ Health. (2021) 9:685141. 10.3389/fpubh.2021.685141 34026721 PMC8131679

[61] Gong X Hou M Han Y Liang H Guo R. Application of the internet platform in monitoring Chinese public attention to the outbreak of COVID-19. Front Publ health. (2021) 9:755530. 10.3389/fpubh.2021.755530 35155335 PMC8831856

[62] Mavragani A Ochoa G. Google Trends in infodemiology and infoveillance: methodology framework. JMIR Publ Health Surveill. (2019) 5:e13439. 10.2196/13439 31144671 PMC6660120

[63] Getis A Ord K. The analysis of spatial association by use of distance statistics. Geograph Anal. (1992) 24:189–206. 10.1111/j.1538-4632.1992.tb00261.x

[64] Wang JF Li XH Christakos G Liao YL Zhang T Gu X. Geographical detectors-based health risk assessment and its application in the neural tube defects study of the Heshun Region, China. Int J Geogr Inform Sci. (2010) 24:107-127. 10.1080/13658810802443457

